# Seroprevalence and risk factors of HBV/HCV/HIV triple co-infection among HIV patients receiving HAART at a Tertiary Hospital in Makurdi, Nigeria

**DOI:** 10.11604/pamj.2026.54.15.52097

**Published:** 2026-05-19

**Authors:** Daniel Olagoke Aina, Shawon Fredrick Akpagher, Blessing Onyinye Kene-Okeke, Abisola Mercy Olowofeso, Anthony Chidiebere Ohanu, Moses Inedu, John Joel Iji, Yanmeer Simeone Tyotswam

**Affiliations:** 1 Acute Medicine, Leicester Royal Infirmary, University Hospitals of Leicester, Leicester, United Kingdom,; 2 Department of Medicine and Surgery, University of Jos, Jos, Plateau State, Nigeria,; 3 Department of Internal Medicine, Benue State University Teaching Hospital, Makurdi, Nigeria,; 4 School of Integrated Sciences, Sustainability and Public Health, University of Illinois Springfield, Illinois, USA,; 5 Public Health Department, University Hospital Limerick, Limerick, Ireland,; 6 Department of Orthopedics, National Orthopedic Hospital, Jos, Plateau State, Nigeria,; 7 Milken Institute School of Public Health, The George Washington University, Washington, D.C., USA,; 8 Department of Population, Reproductive Health and Community Resources Management, Kenyatta University, Kenyatta, Kenya

**Keywords:** HIV infections, hepatitis B virus, hepatitis C virus, coinfection, risk factors, Nigeria

## Abstract

**Introduction:**

co-infection of hepatitis B and C viruses with HIV is associated with poorer survival, increased risk of severe liver disease, and greater highly active antiretroviral therapy (HAART)-related liver toxicity. This study assessed the seroprevalence and risk factors for triple co-infection with hepatitis B virus, hepatitis C virus, and human immunodeficiency virus (HBV/HCV/HIV) among HIV-positive patients on HAART at the Federal Medical Centre, Makurdi.

**Methods:**

in this cross-sectional study, 250 HIV-positive adults were recruited consecutively over three months. Socio-demographic data and risk factors were collected using structured questionnaires. Serum samples were analyzed for hepatitis B and C. Prevalence was calculated, and associations with triple co-infection were examined using chi-square and logistic regression analyses (SPSS v27.0, 95% confidence level).

**Results:**

the prevalence of HBV/HCV/HIV triple co-infection was 1.2% among 250 HIV patients receiving HAART. The cohort was predominantly female (74.4%), with a female-to-male ratio of 3: 1 and a mean age of 45 years. Secondary education (AOR = 3.080, 95% CI: 1.420-7.038, p = 0.020), multiple sexual partners (AOR = 2.200, 95% CI: 1.271-7.073, p = 0.001), and smoking (AOR = 4.071, 95% CI: 1.101-11.018, p = 0.001) were independent predictors of triple co-infection. No significant associations were observed with sex, age, marital status, employment, alcohol use, or body mass index (BMI).

**Conclusion:**

the 1.2% seroprevalence of triple co-infection is low but highlights the ongoing presence of these infections. This finding reinforces the need for stronger public health education and more targeted screening programs. These measures can help reduce risky behaviors and encourage early testing to curb transmission.

## Introduction

Chronic infections caused by hepatitis B virus (HBV), hepatitis C virus (HCV), and human immunodeficiency virus (HIV), the causative agent of acquired immunodeficiency syndrome (AIDS), remain major global public health challenges, collectively accounting for substantial morbidity and mortality worldwide. Recent estimates indicate that approximately 254 million people are living with chronic HBV infection and nearly 50 million with chronic HCV infection, while about 40.8 million individuals were living with HIV globally by the end of 2024 [1].

Sub-Saharan Africa bears a disproportionate share of this burden, accounting for more than two-thirds of global HIV infections, with an estimated adult prevalence of 3.1%. The region also experiences a high prevalence of chronic HBV infection and HIV-HBV coinfection, contributing significantly to virally mediated chronic liver disease and its complications [2]. Because HBV, HCV, and HIV share common routes of transmission, particularly exposure to infected blood and sexual contact, coinfection is frequent, and in some individuals, simultaneous infection with all three viruses may occur [3]. In high-income countries, both HBV and HCV infections are markedly more prevalent among people living with HIV (PLWHIV) than in the general population [3].

However, the epidemiology of these infections differs substantially between resource-rich and resource-limited settings. In many African countries, HBV infection is commonly acquired during early childhood, often preceding HIV acquisition, whereas HCV transmission patterns vary by healthcare practices and sociocultural factors. As a result, prevalence estimates and patterns of dual and triple viral coinfection reported in Western populations cannot be directly extrapolated to African settings, and the burden of concurrent HBV/HCV/HIV infection remains poorly defined [4].

The scale-up of highly active antiretroviral therapy (HAART) across sub-Saharan Africa has led to substantial improvements in survival and life expectancy among PLWHIV. As this population ages, liver-related morbidity and mortality attributable to chronic viral hepatitis have emerged as increasingly important contributors to long-term clinical outcomes. Evidence indicates that HIV infection accelerates the progression of liver disease associated with both HBV and HCV, leading to more rapid fibrosis, cirrhosis, and hepatic dysfunction [5].

These effects may be further amplified in individuals with triple HBV/HCV/HIV infection, in whom overlapping viral replication, immune dysregulation, and cumulative hepatotoxicity may pose additional clinical challenges. Moreover, triple infection complicates antiretroviral treatment selection, particularly in settings where agents with dual anti-HIV and anti-HBV activity, such as tenofovir, are widely used, and therapeutic options are limited.

Despite the high endemicity of HBV, HCV, and HIV in sub-Saharan Africa, data describing the prevalence, demographic characteristics, and clinical implications of triple viral infections remain scarce. Little is known about the immunologic and virologic profiles of individuals with concurrent HBV, HCV, and HIV infection, or the extent to which triple infection influences disease progression and clinical outcomes in routine care settings. Addressing these gaps is essential to inform targeted screening strategies, optimize antiretroviral and hepatitis treatment approaches, and guide public health policy in high-burden regions. This study aimed to determine the prevalence of triple viral coinfections and to assess the impact of risk factors among HIV-infected individuals receiving HAART at Federal Medical Centre (FMC), Makurdi, Nigeria.

## Methods

**Study area:** the study was conducted at the AIDS Prevention Initiative in Nigeria (APIN) Outpatient Clinic of the Federal Medical Centre Makurdi, located in Makurdi, the capital of Benue State, in North-Central Nigeria. FMC Makurdi is a leading tertiary healthcare facility in the region and serves a large population of people living with HIV (PLWHIV). Makurdi is a cosmopolitan city with significant cultural diversity and a well-documented HIV burden. The presence of a robust HIV treatment program makes FMC Makurdi an important and appropriate site for this study.

**Study design:** this was a hospital-based cross-sectional study involving HIV-positive adults aged 18 to 80 years. Participants were recruited from the APIN Antiretroviral Therapy Clinic at FMC Makurdi. Recruitment was conducted over a three-month period, from September to November 2023.

**Study population:** participants were recruited from HIV-infected patients who came to the center for drug refill or routine check-up.

**Inclusion and exclusion criteria:** participants were those with confirmed HIV/AIDS status (via Western blot), either symptomatic or asymptomatic, and receiving ART for at least two years. Patients with medical conditions that could interfere with study outcomes, such as chronic liver disease, severe anemia, pregnancy, or neurological conditions that may impair cognitive recall, were excluded.

**Sampling technique:** a consecutive sampling technique was used to enroll HIV patients on HAART from the outpatient clinic on the study site.

**Administration of questionnaires:** socio-demographic characteristics, medical history, height, and weight were collected using a structured self-administered questionnaire; the type of drug regimen and the duration of treatment were also considered.

**Blood specimen collection:** about 2 ml of venous blood sample was collected from each participant into a labelled dry tube using a Vacutainer needle. Samples were allowed to clot and then centrifuged at 3000 rpm for 10 minutes to obtain serum, which was preserved at standard temperature and later used for screening. HBV and HCV infections were tested using serum samples and HBV and HCV reagent packs.

**Statistical analysis:** chi-square analysis was used to test the significant association between sociodemographic characteristics and risk factors with HBV/HCV/HIV triple co-infection. Binary logistic regression was used to test the independent predictors of HBV/HCV/HIV co-infection, using Statistical Package for Social Sciences (SPSS) version 27.0. All statistical tests were carried out at a 95% confidence level. Results were presented in tables.

**Ethical clearance:** the study was approved by the Health Research Ethical Committee (HREC) of Federal Medical Center, Makurdi, Benue State, with Ref. No: FMH/FMC/HREC/108/VL.1. Study participation was preceded by the written informed consent of each participant, after a thorough explanation and clarification of study aims. Participation in the study was voluntary, with confidentiality and anonymity of study participants assured.

## Results

The sociodemographic characteristics of the 250 study participants are summarized in Table 1. Females comprised 74.4% of respondents and males 25.6%, corresponding to a female-to-male ratio of approximately 3:1. The majority of respondents (52.8%) were between 41 and 60 years old, with a mean age of 44.9 (±11.7) years. Married participants accounted for 55.6% of the sample. Secondary education was the most common educational level (38.0%), followed by tertiary education (28.4%), no formal education (21.2%), and primary education (12.4%). Regarding employment, 32.0% were employed, and 68.0% were unemployed (Table 1).

The behavioral risk-factor profile of participants is presented in Table 2. Multiple sexual partners (MSPs) were reported by 34.8% of respondents, and 46.4% admitted to sharing sharps. Alcohol consumption was reported by 38.0%, while only 6.8% were smokers. In terms of body mass index (BMI), 52.4% of participants were in the normal range, 24.0% were overweight, 10.4% moderately obese, 6.0% severely obese, 1.6% morbidly obese, and 5.6% underweight (Table 2).

**Table 1:** socio-demographic characteristics of 250 HIV-positive patients receiving HAART at the APIN Outpatient Clinic, Federal Medical Centre Makurdi, Benue State, Nigeria, September-November 2023 (N=250)

Variables	Response	Frequency	Percentage (%)
Sex	Male	64	25.6
	Female	186	74.4
	Sex ratio (M: F)		1:3
Age (years)	≤20	2	0.8
	21-40	95	38.0
	41-60	132	52.8
	61-80	21	8.4
	Mean age		44.9±11.7
Religion	Christianity	247	98.8
	Islam	3	1.2
Marital Status	Single	31	12.4
	Married	139	55.6
	Widowed	69	27.6
	Divorced	11	4.4
Level of education	NFE	53	21.2
	Primary	27	10.8
	Secondary	88	35.2
	Tertiary	129	51.6
Employment	Employed	80	32.0
	Unemployed	170	68.0

NFE: no formal education; HAART: highly active antiretroviral therapy; APIN: AIDS Prevention Initiative in Nigeria

**Table 2:** behavioral characteristics of 250 HIV-positive patients receiving HAART at the APIN Outpatient Clinic, Federal Medical Centre Makurdi, Benue State, Nigeria, September-November 2023 (N=250)

Variables	Response	Frequency	Percentage (%)
MSP`s	Yes	87	34.8
	No	163	65.2
Sharing of sharps	Yes	116	46.4
	No	134	53.6
Alcohol use	Yes	95	38.0
	No	155	62.0
Smoking	Yes	17	6.8
	No	233	93.2
BMI (kg/m^2^)	Underweight	14	5.6
	Normal	131	52.4
	Overweight	60	24.0
	Moderately obese	26	10.4
	Severely obese	15	6.0
	Morbidly obese	4	1.6

MSP: multiple sexual partners; BMI: body mass index; HAART: highly active antiretroviral therapy; APIN: AIDS Prevention Initiative in Nigeria

The overall prevalence of HBV/HCV/HIV triple co-infection was 1.2%, as shown in Table 3. Level of education was the only sociodemographic variable significantly associated with triple co-infection (p = 0.023): prevalence was 2.1% among participants with secondary education and 1.4% among those with tertiary education, with no cases observed among individuals with no formal or primary education. Sex, age group, religion, marital status, and employment status showed no significant associations (all p > 0.05) (Table 3).

**Table 3:** prevalence of HBV/HCV/HIV triple co-infection by sociodemographic characteristics among 250 HIV-positive patients receiving HAART at the APIN Outpatient Clinic, Federal Medical Centre Makurdi, Benue State, Nigeria, September-November 2023 (N=250)

			Triple co-infection (%)			
Variables	Response	Freq.	Positive	Negative	X^2^	p-value
Sex	Male	64	2 (3.1)	62 (96.9)	2.689	0.101
	Female	186	1 (0.5)	185 (99.5)		
	Total	250	3 (1.2)	247 (98.8)		
Age (years)	≤20	2	-	2 (100)	0.407	0.939
	21-40	95	1 (1.1)	94 (98.9)		
	41-60	132	2 (1.5)	130 (98.5)		
	61-80	21	-	21 (100)		
	Total	250	3 (1.2)	247 (98.8)		
Religion	Christianity	247	3 (1.2)	244 (98.8)	0.037	0.848
	Islam	3	-	3 (100)		
	Total	250	3 (1.2)	247 (98.8)		
Marital Status	Single	31	1 (3.2)	30 (96.8)	2.112	0.550
	Married	139	2 (1.4)	137 (98.6)		
	Widowed	69	-	69 (100)		
	Divorced	11	-	11 (100)		
	Total	250	3 (1.2)	247 (98.8)		
Level of education	NFE	53	-	53 (100)	6.217	0.023*
	Primary	31	-	31 (100)		
	Secondary	95	2 (2.1)	93 (97.9)		
	Tertiary	71	1 (1.4)	70 (98.6)		
	Total	250	3 (1.2)	247 (98.8)		
Employment	Unemployed	80	1 (1.3)	79 (98.8)	0.222	0.960
	Employed	170	2 (1.2)	168 (98.8)		
	Total	250	3 (1.2)	247 (98.8)		

* p < 0.05 (statistically significant); NFE: no formal education; HAART: highly active antiretroviral therapy; APIN: AIDS Prevention Initiative in Nigeria; HBV/HCV/HIV: hepatitis B virus/hepatitis C virus/human immunodeficiency virus

The associations between behavioral risk factors and triple co-infection status are presented in Table 4. Participants with multiple sexual partners had a significantly higher co-infection rate (3.4%) compared with those without (0%; p = 0.017). Sharing of sharps was also associated with increased co-infection risk: 2.6% among those who shared sharps versus 0% among those who did not (p = 0.026). Smoking demonstrated a particularly strong association, with 11.8% of smokers testing positive compared with only 0.4% of non-smokers (p = 0.001). No significant associations were found between co-infection and alcohol use (p = 0.479) or BMI (p = 0.967) (Table 4).

**Table 4:** prevalence of HBV/HCV/HIV triple co-infection by behavioral risk factors among 250 HIV-positive patients receiving HAART at the APIN Outpatient Clinic, Federal Medical Centre Makurdi, Benue State, Nigeria, September-November 2023 (N=250)

			HBV/HCV/HIV triple co-infection (%)			
Variables	Response	Freq.	Negative	Positive	X^2^	p-value
MSP`s	Yes	87	3 (3.4)	84 (96.6)	5.689	0.017*
	No	163	-	163 (100)		
	Total	250	3 (1.2)	247 (98.8)		
Alcohol use	Yes	95	2 (2.1)	93 (97.9)	0.501	0.479
	No	155	1 (0.6)	154 (99.4)		
	Total	250	3 (1.2)	247 (98.8)		
Sharing of sharps	Yes	116	3 (2.6)	113 (97.4)	4.954	0.026*
	No	134	-	134 (100)		
	Total	250	3 (1.2)	247 (98.8)		
Smoking	Yes	17	2 (11.8)	15 (88.2)	17.172	0.001*
	No	233	1 (0.4)	232 (99.6)		
	Total	250	3 (1.2)	247 (98.8)		
BMI	Underweight	14	-	14 (100)	0.945	0.967
	Normal	131	2 (1.5)	129 (98.5)		
	Overweight	60	1 (1.7)	59 (98.3)		
	Moderately obese	26	-	26 (100)		
	Severely obese	15	-	15 (100)		
	Morbidly obese	4	-	4 (100)		
	Total	250	3 (1.2)	247 (98.8)		

* p < 0.05 (statistically significant); MSP: multiple sexual partners; BMI: body mass index; HBV/HCV/HIV: hepatitis B virus/hepatitis C virus/human immunodeficiency virus; HAART: highly active antiretroviral therapy; APIN: AIDS Prevention Initiative in Nigeria

Multivariate logistic regression results identifying independent predictors of triple co-infection are presented in Table 5. Participants with secondary-level education had significantly higher odds of triple co-infection compared with those with tertiary education (AOR = 3.080, 95% CI: 1.420-7.038, p = 0.020). Multiple sexual partnerships were independently associated with increased odds (AOR = 2.200, 95% CI: 1.271-7.073, p = 0.001). Smoking was the strongest predictor, with smokers nearly four times more likely to have triple co-infection (AOR = 4.071, 95% CI: 1.101-11.018, p = 0.001). Although sharing of sharp objects was associated with elevated odds (AOR = 3.221, 95% CI: 0.981-9.002), this association did not retain statistical significance after adjustment (p > 0.05) (Table 5).

**Table 5:** independent predictors of HBV/HCV/HIV triple co-infection among 250 HIV-positive patients receiving HAART at the APIN Outpatient Clinic, Federal Medical Centre Makurdi, Benue State, Nigeria, September-November 2023 (N=250)

				95% C.I (AOR)	
Predictors	Response	AOR	p-value	Lower	Upper
LOE	NFE	1.114	0.117	0.245	8.456
	Primary	2.015	>0.05	0.719	7.281
	Secondary	3.080	0.020*	1.420	7.038
	Tertiary (Ref)	1.000			
MSP`s	Yes	2.200	0.001*	1.271	7.073
	No	1.000			
Sharing of sharps	Yes	3.221	>0.05	0.981	9.002
	No (Ref)	1.000			
Smoking	Yes	4.071	0.001*	1.101	11.018
	No	1.000			

* p < 0.05 (statistically significant); LOE: level of education; MSP: multiple sexual partners; NFE: no formal education; AOR: adjusted odds ratio; CI: confidence interval; Ref: reference category; HBV/HCV/HIV: hepatitis B virus/hepatitis C virus/human immunodeficiency virus; HAART: highly active antiretroviral therapy; APIN: AIDS Prevention Initiative in Nigeria

## Discussion

The present study recorded a prevalence of HBV/HCV/HIV triple co-infection of 1.2% among 250 HIV patients receiving HAART, characterized by a predominantly female cohort (74.4%), a female-to-male sex ratio of 3:1, and a mean age of 45 years. Secondary level of education, multiple sexual partners, and smoking were identified as independent predictors of triple co-infection, while sociodemographic factors, including sex, age, marital status, employment, alcohol use, and BMI, showed no significant associations. These findings could be attributed to several interrelated factors, such as the shared transmission routes of the three viruses, primarily through unprotected sexual practices and sharing needles and other sharp objects, as well as low education and awareness, which often correlate with poor health-seeking behaviors and limited knowledge of preventive measures [6].

The female sex preponderance is consistent with national figures showing that more than half of people living with HIV/AIDS (PLWHA) in Nigeria are women, whether in rural or urban areas [6,7]. The study's mean age of 45 years aligns with national statistics, indicating that the burden of HIV infection in Nigeria is highest among individuals aged 15-49 years, who represent approximately 75% of all PLWHA [7]. The higher representation of women may also reflect greater clinic attendance during the study period [6].

The triple co-infection rate of 1.2% is substantially low and aligns with national estimates ranging from 0.7% to 1.3% in studies of HIV-positive populations [8]. Global estimates for triple co-infection range from 0.3% to 3.5% [9]. The observed rate is consistent with a systematic review from Nepal that also reported a 1.3% prevalence [10]. A separate study in Nepal recorded a lower rate of 0.3% [11], while a Brazilian study reported 0.16% [12]. Notably, some high-income countries have reported rates exceeding the present finding. A study in New York City reported a prevalence of 1.58% [13], attributed largely to intravenous drug use (IDU), which represents a shared blood-borne transmission pathway for all three viruses. Higher rates have also been reported in Hunan Province, China (2.72%) [14] and Nepal (2.53%) [9].

In Africa, a systematic review documented a pooled triple co-infection prevalence of 0.7% across 314 studies [4]. Despite this pooled figure, substantial heterogeneity exists: in Ghana, prevalence estimates have ranged from 0.0% [15] to 18.0% [16]; Kenya reported 0.0% [17]; and both Uganda and Ethiopia reported 1.1% [18,19]. Based on these comparisons, the prevalence recorded in this study is consistent with multiple other African studies, yet lower than that reported in several high-income countries. This disparity likely reflects differences in dominant HIV transmission routes: in many African countries, transmission occurs primarily via sexual contact, whereas parenteral exposure, especially IDU, contributes substantially to co-infection rates in high-income settings.

Within Nigeria, similar triple co-infection rates have been reported: 1.3% in Ikole, Ekiti State [20]; 1.5% and 0.64% in Abuja [21,22]; 1.0% in Calabar [23]; 0.8% in Port Harcourt [24]; 0.6% in Benin City [25]; 0.58% in Nsukka, Enugu State [26]; and 1.0% in Bauchi, North-East Nigeria [27]. Notably higher prevalences have been recorded in North-Central and North-West Nigeria, with 7.2% in Keffi, Nasarawa State, and 1.9% in Maiduguri, Borno State, highlighting marked regional heterogeneity [28,29].

The clinical significance of triple co-infection deserves emphasis. Concurrent HBV/HCV/HIV infection may exacerbate immunosuppression, promote higher levels of viral hepatitis replication, reduce the likelihood of spontaneous clearance of HBV and HCV, and increase the risk of reactivation. Individuals with HBV or HCV co-infection may also be more susceptible to hepatotoxicity associated with antiretroviral therapy or immune reconstitution. Collectively, these factors elevate the risk of progressive liver disease, including cirrhosis [6,30,31].

Regarding educational attainment, participants with secondary-level education had the highest prevalence of triple co-infection (2.1%), compared with 1.4% among those with tertiary education. Those with secondary education were approximately three times more likely to have triple co-infection than those with tertiary education. Low educational attainment, which correlates with poor health-seeking behaviors and limited awareness of preventive measures, likely contributed to this pattern [6,32]. A study in a comparable Nigerian setting similarly found lower educational status to be significantly associated with co-infection rates [33].

Although sharing of sharp objects was associated with increased odds of triple co-infection (AOR = 3.221), this association did not reach statistical significance after adjustment (95% CI: 0.981-9.002), possibly because of the small number of triple-infection cases limiting statistical power. Smoking emerged as an independent predictor, nearly quadrupling the odds of triple co-infection. Both smoking and alcohol use may contribute to behaviors that elevate the risk of exposure to blood-borne viruses, including unprotected sexual activity and the use of non-sterile injecting equipment. This is consistent with prior research [6,9,13]. Having multiple sexual partners was significantly associated with co-infection status and increased the odds by approximately two-fold, aligning with previous studies [13,34].

**Limitations:** the absence of significant associations for sex, age, religion, marital status, employment, alcohol, and BMI may partly reflect study limitations. Reliance on self-reported questionnaires may have introduced social desirability bias. Recruitment from a single tertiary hospital limits generalizability to all HIV patients in Makurdi, one of three major tertiary centers in the area. Additionally, the immunosuppressive effect of HIV on responses to HBV infection may independently increase HBV acquisition risk, independent of the behavioral factors assessed.

## Conclusion

The observed prevalence of 1.2% highlights the ongoing co-circulation of HBV, HCV, and HIV despite existing public health interventions and underscores the need for routine hepatitis screening prior to initiating HAART in HIV-positive patients. This study identifies secondary-level education, multiple sexual partners, and smoking as independently associated with triple co-infection. Targeted public health interventions, including health education campaigns, harm reduction programs, and integration of routine HBV and HCV screening within HIV treatment platforms, are warranted. Further multicenter research is needed to inform effective prevention, monitoring, and treatment strategies for individuals with multiple viral infections in Nigeria.

### What is known about this topic

- Hepatitis B virus and HCV coinfections are common among people living with HIV in sub-Saharan Africa;
- Viral hepatitis coinfection accelerates liver disease progression and increases liver-related complications in HIV patients;
- Behavioral risk factors contribute to the transmission of blood-borne viral infections.

### What this study adds

- This study provides updated evidence on HBV/HCV/HIV triple co-infection among HIV patients receiving HAART in Makurdi, Nigeria;
- Multiple sexual partners and smoking were identified as significant predictors of triple co-infection;
- The findings highlight the need to strengthen routine viral hepatitis screening within HIV care programs.
